# The effect of post-reperfusion levosimendan in an experimental intestinal ischemia–reperfusion model

**DOI:** 10.1186/s44158-022-00074-3

**Published:** 2022-10-27

**Authors:** Hakan Aygun, Cimen Olguner, Ugur Koca, Bekir Ugur Ergur, Ali Rıza Sisman, Duyguhan Isguven, Pelin Girgin, Muhammed Akkus, Serkan Tulgar

**Affiliations:** 1 Department of Anesthesiology, Bakircay University Cigli Training and Research Hospital, 8780/1 Sokak No:18 Yeni Mahalle Ata Sanayi, Izmir, Turkey; 2 Department of Anesthesiology, Dokuz Eylul University Faculty of Medicine, Izmir, Turkey; 3 Department of Histology and Embryology, Dokuz Eylul University Faculty of Medicine, Izmir, Turkey; 4 Department of Medical Biochemistry, Dokuz Eylul University Faculty of Medicine, Izmir, Turkey; 5 Department of Anesthesiology, Samsun University, Samsun Training and Research Hospital, Samsun, Turkey

**Keywords:** Phosphodiesterase 3 inhibitors, Ischemia, Reperfusion, Rats, Mesenteric vascular occlusion, Simendan

## Abstract

**Background:**

Levosimendan has been reported to have a positive effect on ischemia–reperfusion injury. Herein, we aimed to evaluate the effects of levosimendan applied after reperfusion in an experimental intestinal injury-reperfusion (IR) model.

**Methods:**

Twenty-one Wistar-albino male rats were separated into three groups: Sham group (*n* = 7): solely superior mesenteric artery (SMA) was dissected after laparotomy; intestinal ischemia–reperfusion group (IIR, *n* = 7): SMA was clamped for 60 min and unclamped for 120 min to cause ischemia–reperfusion; IIR + levosimendan group (IIR + L, *n* = 7): levosimendan was administered in ischemia–reperfusion model. The mean arterial pressures (MAP) were measured in all groups. MAP measurements were performed at the end of stabilization, at the 15th, 30th, and 60th minute of ischemia; at the 15th, 30th, 60th, and 120th minute of reperfusion; and at the end of levosimendan bolus application and when levosimendan infusion concluded. Reperfusion injury was evaluated with tissue malondialdehyde (MDA) and by Chiu score.

**Results:**

MAP at 15 min, 30 min, and 60 min of reperfusion was lower in IIR and IIR + L groups compared with basal inter-group measurements. Decline in MAP at 30 min after reperfusion was statistically significant in IIR and IIR + L groups when compared with the sham group. There was no significant difference between MDA levels in the groups. Chiu score was significantly lower in the sham group when compared to IIR and IIR + L groups and higher in IIR when compared to the IIR + L group.

**Conclusion:**

Levosimendan leads to a decrease in intestinal damage although it did not affect lipid peroxidation and MAP when administered after reperfusion in an experimental intestinal IR model.

## Introduction

Pathological changes that occur after ischemia and reperfusion periods in the intestines are called intestinal ischemia–reperfusion damage [1]. Intestinal ischemia is a serious and frequent clinical condition that occurs as a result of a decreased or complete cessation of intestinal arterial blood flow. This leads to severe local or extensive tissue damage [1, 2]. However, reperfusion in ischemic tissues leads to more damage than ischemia alone [3]. A decrease in arterial or venous blood flow leads to oxygen deprivation of tissues or organs, depletion of cellular energy stores, and accumulation of toxic metabolites followed by reversible or irreversible cell and tissue damage that may lead to cell death. There are two basic mechanisms that lead to reperfusion injury: the first is the release of free oxygen radicals (SOR), and the second is the activation of phospholipase A2 by the release of calcium (Ca + +) during the ischemic period leading to a breakdown of fatty acids in the membranes [1, 3, 4]. Damage caused by ischemia–reperfusion is not only limited locally, but it may also cause damage to distant organs such as the lungs, liver, heart, brain, and kidneys leading to multiple organ failure (MOF) due to many activated mechanisms and toxic by-products [4].

Levosimendan is a cardiovascular drug with positive inotropic and vasodilator features, approved for the treatment of acute and decompensated heart failure. It causes increased sensitivity of troponin C to calcium, leading to a positive inotropic effect. It also has a vasodilator and anti-ischemic effect due as it leads to the opening of adenosine triphosphate (ATP)-sensitive potassium (K +) channels and phosphodiesterase III inhibition. Promising data on levosimendan in ischemia–reperfusion injury by the effects on potassium (K +) channels in myocardial, intestinal, hepatic, and pulmonary cells are presented profusely [5]. Levosimendan binds to proteins at a rate of 95–98%. It is metabolized through conjugation in the liver, and 1/3 is excreted through urine and 1/3 through feces. However, bacteria in the gut reduce it to the active metabolite OR-1855 which is an aromatic amine. OR-1855 is reabsorbed in the liver and metabolized to a more active OR-1896 form. The half-life of OR-1896 in healthy volunteers was reported as 60 h [6].

Since the intestines play an important role in systemic inflammatory response syndrome, the effect of vasoactive agents on splanchnic perfusion is important. One study demonstrated that levosimendan increased oxygen presentation to the intestinal tissue in the septic shock model secondary to endotoxins in pigs [7]. The effect of levosimendan in increasing microvascular gastric mucosal oxygenation has been found to be superior to other inotropes such as dobutamine and milrinone [8]. Özkaya et al. [9] reported that levosimendan reduced the damage caused by IIR in the rat model when levosimendan was given as a pre-treatment. However, levosimendan is not routinely used in the clinical treatment of intestinal ischemia, and that study demonstrated that intestinal injury can be reduced by early administration of levosimendan. We aimed to examine the effects of levosimendan applied after reperfusion in an experimental rat IIR model. We hypothesized that levosimendan had no effect post-reperfusion in an experimental rat IIR model.

## Methods

This randomized control experimental study was performed at Dokuz Eylul University Faculty of Medicine Experimental Animal Laboratory following permission from the local animal experiment ethics committee (date: July 16, 2010, protocol number: 43/2010). In the study, 21 Wistar Albino adult male rats weighing 250–300 g were used. Rats were fed with standard pellet chow and water ad libitum and were kept in wire cages for 12-h cycles of light/dark at room temperature (21–22 °C) and 40–60% relative humidity. They were only allowed to drink water for 12 h leading up to surgery. During the experiments, the body temperature of the animals was maintained with a heating lamp. Anesthesia was provided by administration of intraperitoneal (IP) 50 mg.kg^−1^ ketamine (Ketalar ®, Pfizer Pharma GMBH, Germany) and 10 mg.kg^−1^ xylazine hydrochloride (Alfazin®, 2%, Alfasan International, 3440 AB, Woerden, Holland). The same anesthetic dose was repeated when necessary to ensure rat immobility. ARRIVE guidelines have been used in this study.

### Groups and protocol

**Fig. 1:**
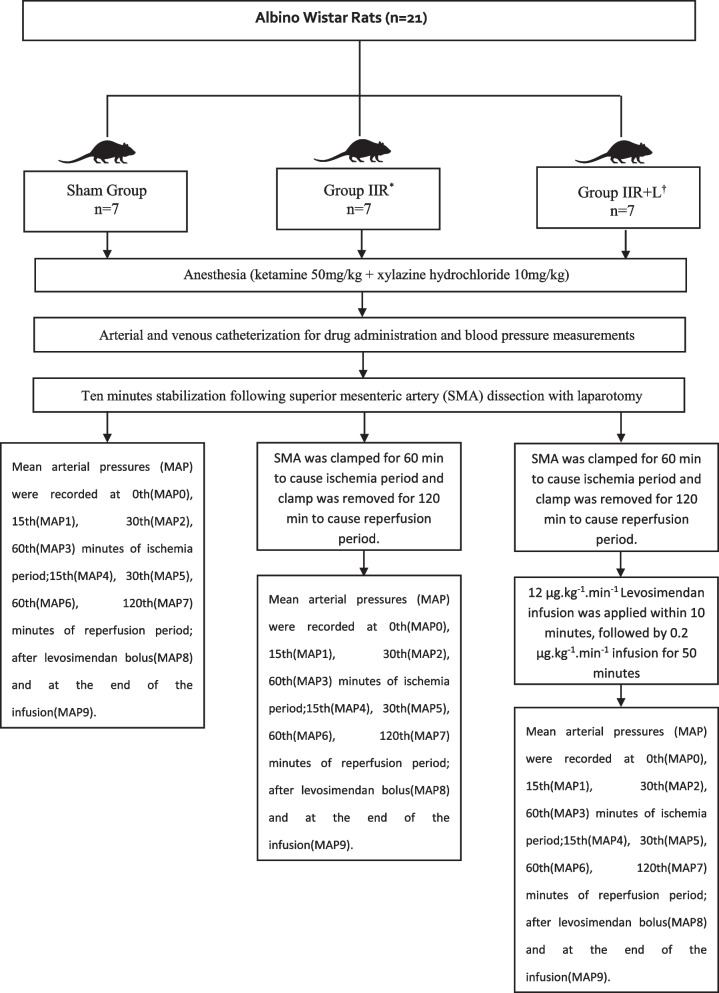
Study groups flow chart. ^*^IIR intestinal ischemia-reperfusion, ^†^IIR + L intestinal ischemia-reperfusion + levosimendan

Simple randomization was used to assign the rats into three groups. Total working time was 240 min in all groups. The following protocols were applied for each respective group (Fig. 1). Sham group (*n* = 7): After laparotomy, the superior mesenteric artery (SMA) was dissected. Rats were kept under anesthesia with no other intervention; intestinal ischemia–reperfusion (IIR) group (*n* = 7): The SMA was dissected and clamped for 60 min, and then, reperfusion was achieved for 120 min [9]; intestinal ischemia–reperfusion + levosimendan (IIR + L) group (*n* = 7): SMA was dissected and clamped for 60 min, and then, reperfusion was provided for 120 min. After reperfusion, 12 µg.kg^−1^.min^−1^ infusion was applied within 10 min, followed by 0.2 µg.kg^−1^.min^−1^ infusion for 50 min [9, 10].

### Dissection of the superior mesenteric artery and ischemia–reperfusion time

**Fig. 2:**
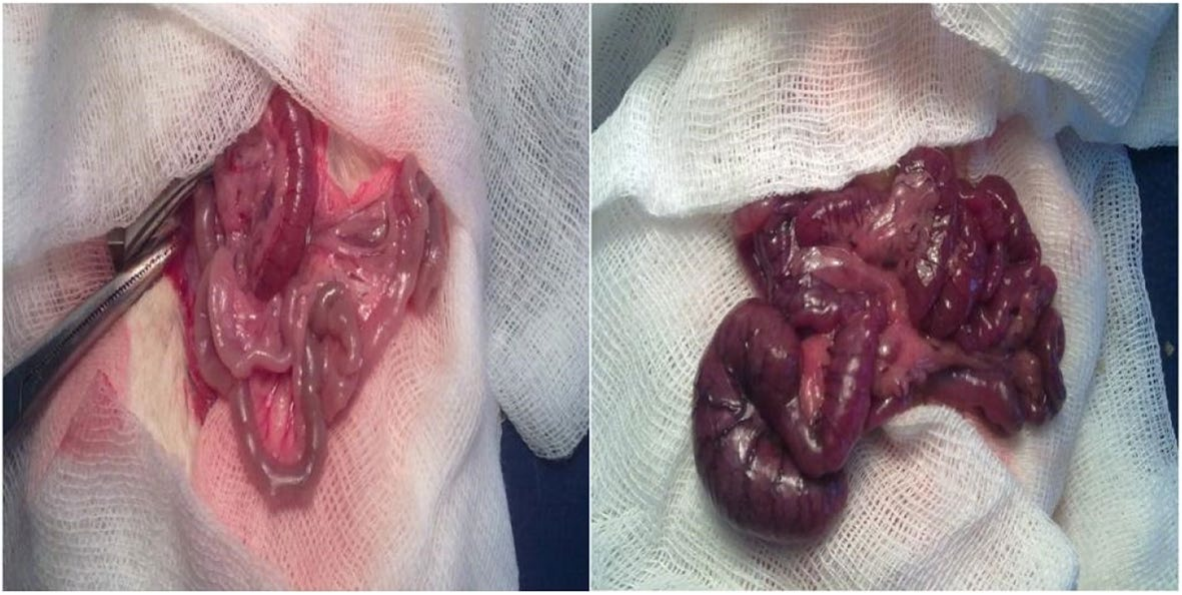
Small intestinal tissue following ischemia and reperfusion

Anesthesia, cannulation, and monitoring were performed followed by a 10-min waiting time for stabilization. Thereafter, 2 mL/h of saline solution infusion was started for all groups. Subsequently, the abdomen was opened with a midline incision under aseptic conditions, the intestines were removed towards the body surface, the Treitz ligament was cut, and the SMA was carefully dissected. Rats in the Sham group were monitored until the end of the experiment after the SMA was dissected. In the rats in the IIR and IIR + L groups, SMA was compressed with an atraumatic microvascular clamp at the exit point from the aorta, and 60-min ischemia was created. Before applying the clamp, 50 IU.kg^−1^ heparin IV was administered to prevent intravascular coagulation. Adequate occlusion was confirmed by the absence of pulsation and pallor in the mesenteric vessels. After 60 min, the clamp was removed and 120 min of reperfusion was provided (Fig. 2). The abdomen was closed with wet sterile tampons during the waiting times.

### Application of experimental drug

At the end of reperfusion, the application of the experimental drug was commenced. Levosimendan (Simdax ® 2.5 mg.mL^−1^, Abbott, Orionpharma, Finland) was administered to the rats in the IIR + L group after preparation of a 2 µg. mL^−1^ concentrate. After an intravenous loading dose of 12 µg.kg^−1^ for 10 min, maintenance infusion at a dose of 0.2 µg.kg^−1^.min^−1^ for 50 min was administered alongside the saline solution. The rats in the Sham and IIR groups were infused with saline solution in the same volume as the study drug applied in the IIR + L group.

### Data recorded during experiments

The mean arterial blood pressure was measured at these time points: At the end of the 10-min stabilization time (MAP-0-basal); at 15 (MAP-1), 30 (MAP-2), 60 (MAP-3) min of ischemia; at 15 (MAP-4), 30 (MAP-5), 60 (MAP-6), and 120 (MAP-7) min of reperfusion; and at the end of levosimendan bolus application (MAP-8) and when levosimendan infusion concluded (MAP-9).

### Collecting of samples and conclusion of experiments

After the administration of levosimendan was completed, a 4-cm section of the proximal terminal ileum was removed from the anesthetized rats, and the intestinal lumen was washed with a cold saline solution. Two centimeters of the removed ileum tissue was stored in 10% buffered formaldehyde for histopathological examination.

The remaining ileum tissue was dried with a surgical pad, then divided into two equal parts for biochemical processes (tissue MDA) and placed in microcentrifuge (Eppendorf) tubes and preserved in a deep freezer at − 80 °C until the day of measurement in the Medical Biochemistry Department.

After the samples were taken, rats were sacrificed with 120 mg.kg^−1^ thiopental IV.

### Histopathological measurements

All histopathological evaluations were performed by a histologist blinded to the groups. The tissues of the ileum were fixed in 10% buffered formaldehyde. After waiting for 24–48 h, the tissues were turned into paraffin blocks for routine histological examination. With the help of a microtome (Leica RM2235, Germany) and a microtome blade (Feather S35), 5-µm-thick sections were taken from the small intestine tissue. The sectional samples taken were placed on slides and stained with hematoxylin–eosin (H&E). Histopathological evaluations of sections obtained from the intestinal tissue were evaluated using the intestinal scoring system defined by Chiu et al. [11] with mucosal lesions graded between 0 and 5 (Table 1).

**Table 1:** Histopathological evaluation of the small intestine according to the Chiu score [11]

Score	Findings
0	Normal mucosal villi
1	Subepithelial separations at the upper end of the villi with capillary congestion
2	Medium-density view with subepithelial separations pushing up the mucous epithelium
3	Subepithelial separations are observed to a large extent, deformations in the villi tips where the mucosal epithelium is intensely pushed upward along the villi
4	Villus deformation reaching the lamina propria with dilated capillaries
5	Lamina propria ulceration, disintegration, and hemorrhage

### Statistical evaluation

Statistical Package of Social Sciences 15 (SPSS 15.0, Chicago, IL, USA) was used for statistical evaluation. Mann–Whitney *U* test was used for paired comparisons of two groups, and Friedman test and Wilcoxon test were used for in-group comparison. All values are shown as mean ± standard deviation (mean ± sd). The statistical value of *p* < 0.05 was considered significant.

## Results

### Mean arterial blood pressure

**Fig. 3:**
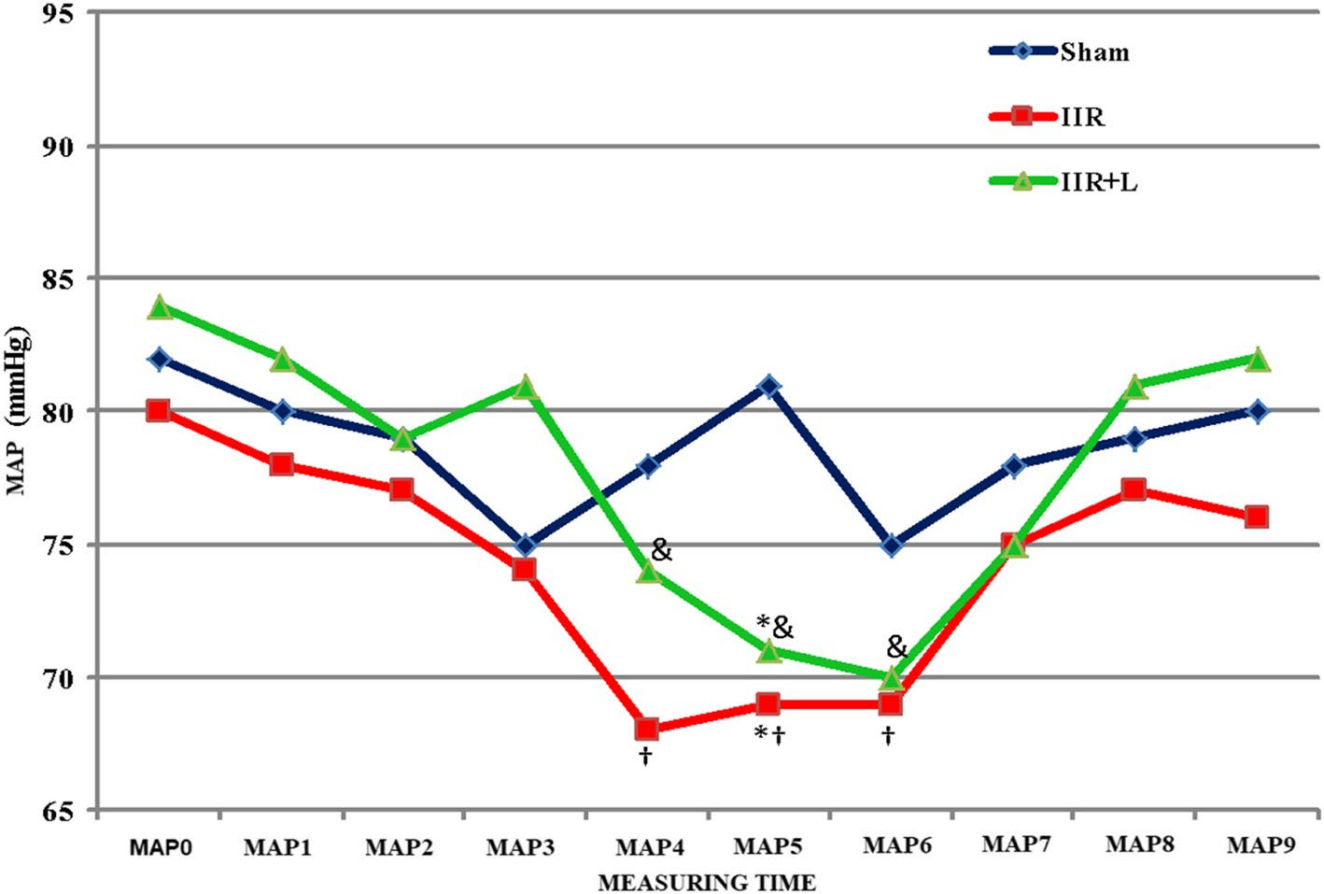
Mean arterial blood pressure levels. *Comparison of IIR and IIR + L groups with sham group (*p* = 0.011, *p* = 0.011). ^&^IIR + L group, group measurements when compared to basal measurements (*p* < 0.001). ^†^IIR group, group measurements when compared to basal measurements (*p* < 0.001). IIR intestinal ischemia-reperfusion, IIR + L intestinal ischemia-reperfusion + levosimendan, MAP mean arterial blood pressure, MAP0 basal time, MAP1 15th min of ischemia, MAP2 30th min of ischemia, MAP3 60th min of ischemia, MAP4 15th min of reperfusion, MAP5 30th min of reperfusion, MAP6 60th min of reperfusion, MAP7 120th min of reperfusion, MAP8 after levosimendan bolus, MAP9 at the end of the levosimendan infusion

Except for MAP-5 (30 min after reperfusion) which is statistically significantly lower in the IIR and IIR + L groups when compared to the sham group (both *p* = 0.011), MAP was similar at all time points in the IIR and IIR + L groups. When time points were evaluated within each group, no statistically significant difference was observed in the sham group. In the IIR and IIR + L groups, there was a significant decrease in MAP at 15-min (MAP-4), 30-min (MAP-5), and 60-min (MAP-6) measurements when compared to the basal measurements (*p* < 0.001). No other significant difference was observed (Fig. 3).

### Tissue malondialdehid (MDA) (µmol/g protein) levels

**Fig. 4:**
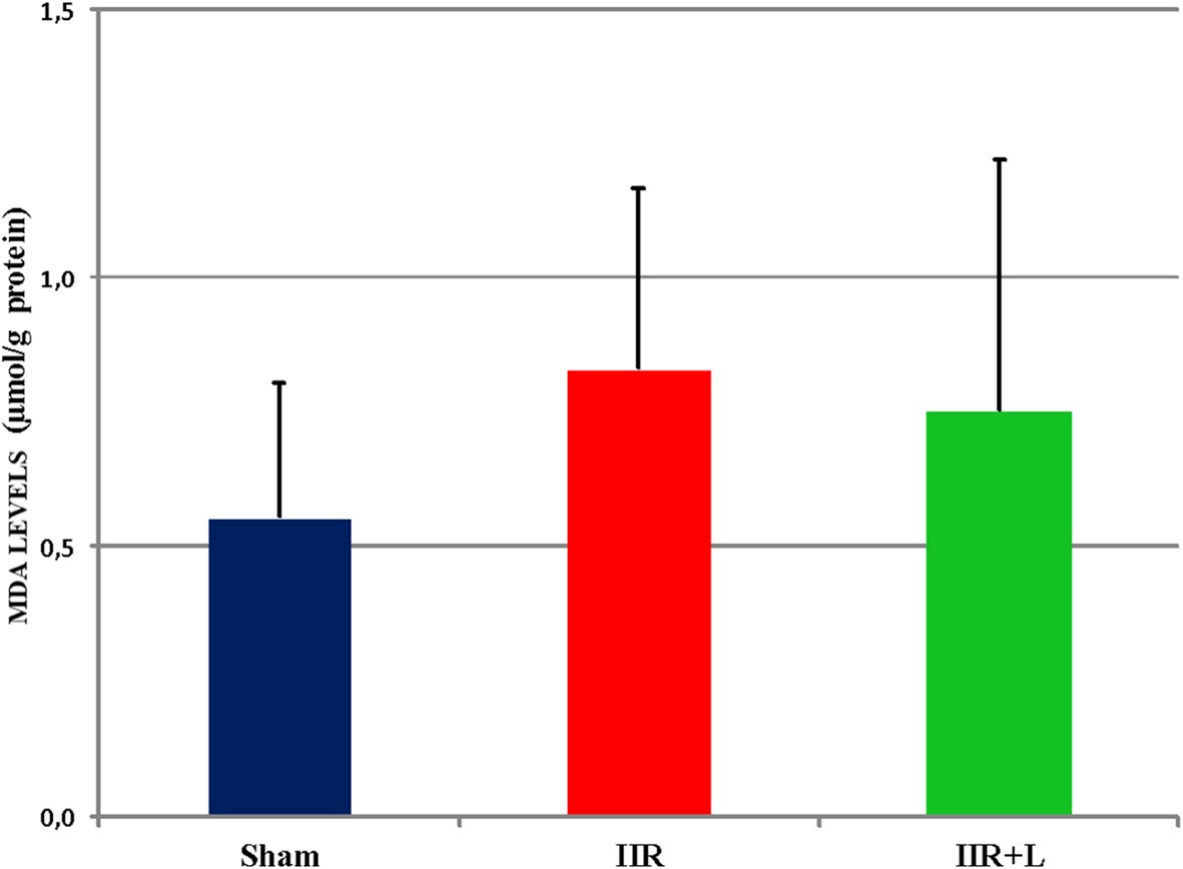
Tissue Malondialdehid (MDA) levels in small intestinal tissue (*p* > 0.05)

No statistically significant difference was seen for MDA levels between groups (Sham 0.555 ± 0.249 vs IIR 0.83 ± 0.338 vs IIR + L 0.752 ± 0.472; *p* = 0.351) (Fig. 4).

### Histopathological findings (Chiu score) (Table 1)

**Fig. 5:**
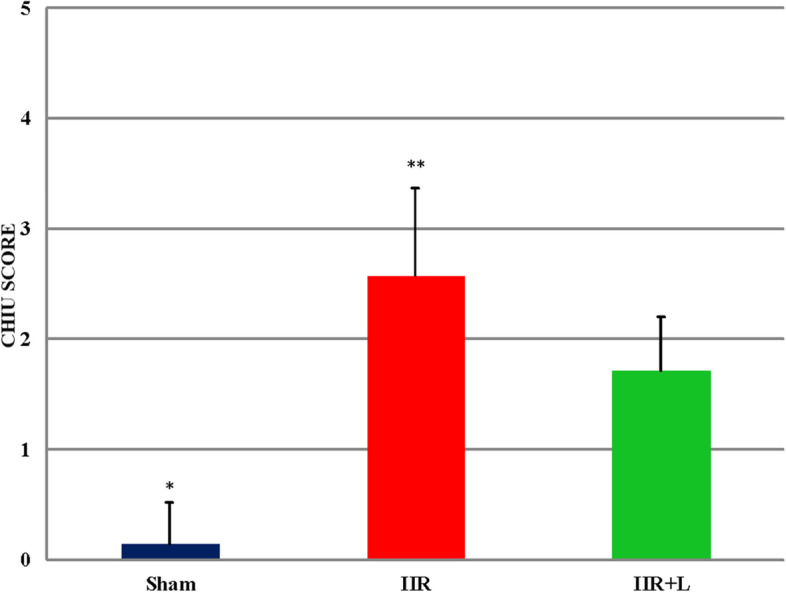
Histopathological evaluation of the small intestinal tissue. *Comparison of the IIR and IIR + L groups with the *Sham* group (*p* = 0.001). **Comparison of the IIR + L group with the IIR group (*p* = 0.03). IIR intestinal ischemia-reperfusion, IIR + L intestinal ischemia-reperfusion + levosimendan

When Chiu scores were compared between groups, scores were statistically significantly lower in the Sham group (0.14 ± 0.38) versus the IIR (2.57 ± 0.79) and IIR + L (1.71 ± 0.49) groups (*p* = 0.001 and *p* = 0.001, respectively). The Chiu score of the IIR group was statistically significantly higher when compared to the IIR + L group (*p* = 0.03) (Fig. 5).

#### In the Sham group

**Fig. 6:**
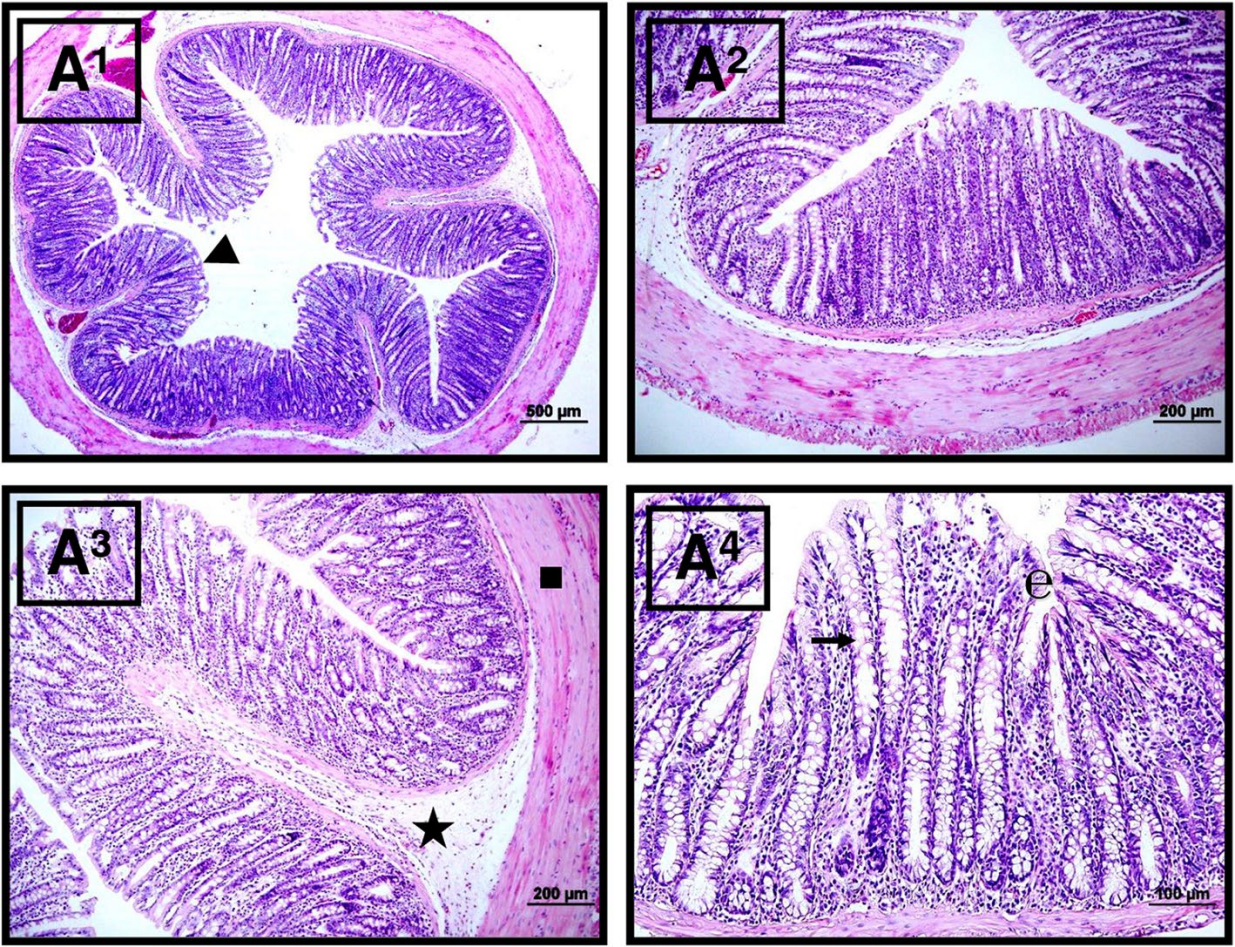
Microscopic view of the *Sham* group tissue samples. Normal small intestinal mucosa: plica circularis (triangle), epithelial layer (**e**), goblet cells (right arrow), submucosal layer (star), and tunica muscularis (square). Hematoxylin–eosin (H&E) stain with amplifications of A1: × 4, A2: × 10, A3: × 20, and A4: × 40)

Microscopic examination of the small intestine sections demonstrated that tissue integrity was normal; morphological changes did not occur in the lamina propria; and ulceration, mononuclear cell infiltration, increased capillary permeability, and hemorrhage were not observed (Fig. 6).

#### In the IIR group

**Fig. 7:**
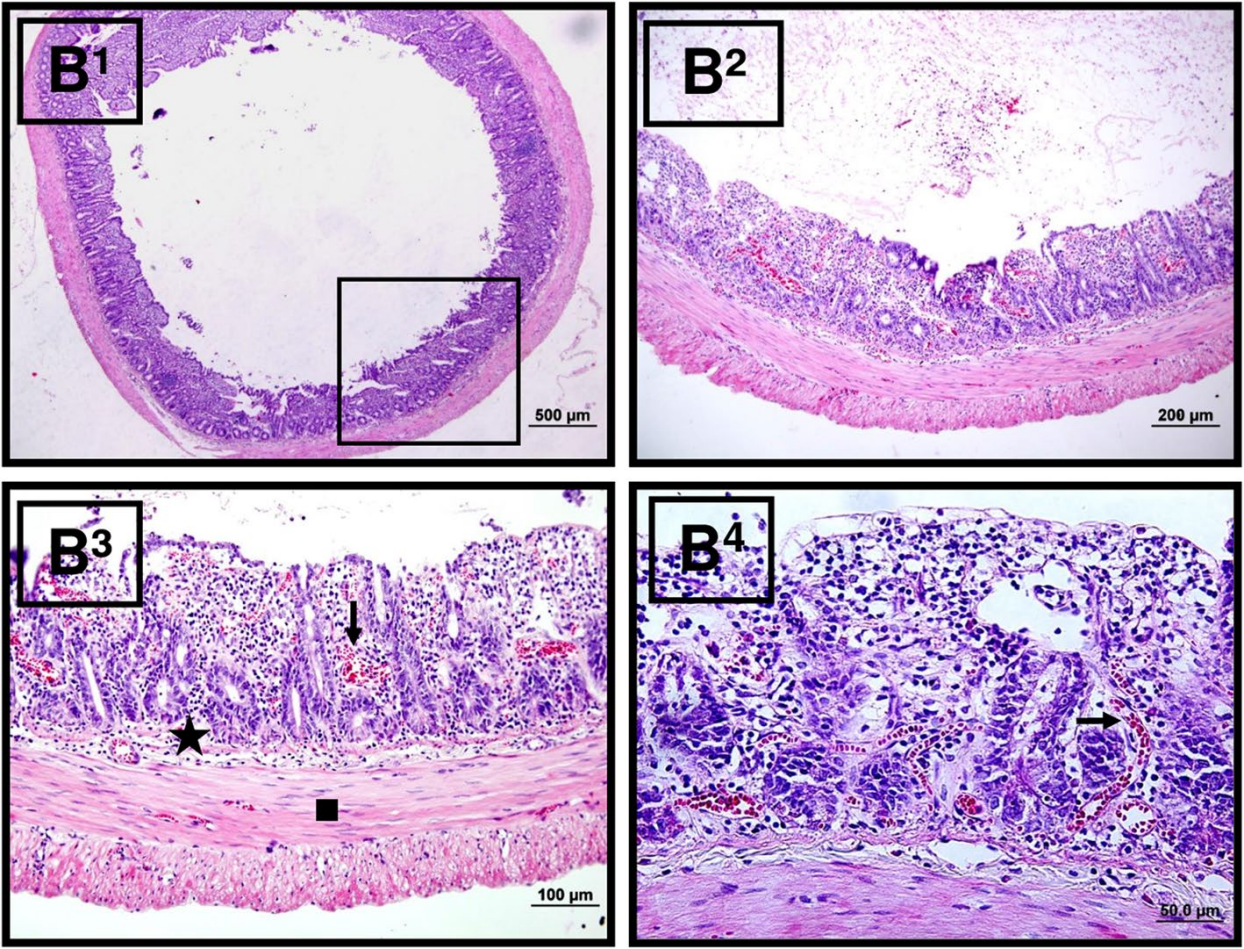
Microscopic view of IIR group tissue samples. Villus degeneration, increase in capillary permeability and congestion (right arrow), increase in mononuclear cells, intraepithelial hemorrhage, and ulcers are observed, and degeneration is observed in the plica circularis and epithelial layers (star) and shows the submucosal layer and the tunica muscularis (square). Hematoxylin–eosin (H&E) stain with amplifications of B1: × 4, B2: × 10, B3: × 20, B4: × 40)

Microscopic examination of the small intestine sections demonstrated that the integrity of the villi was impaired; there was shortening, blunting, and joints in the villi; and a decrease in the number of goblet cells was observed along with the shedding and decrease in the number of crypt epithelial cells. Mononuclear cell infiltration, increased capillary permeability, and hemorrhage were observed in the lamina propria (Fig. 7).

#### *In the IIR* + *L group*

**Fig. 8:**
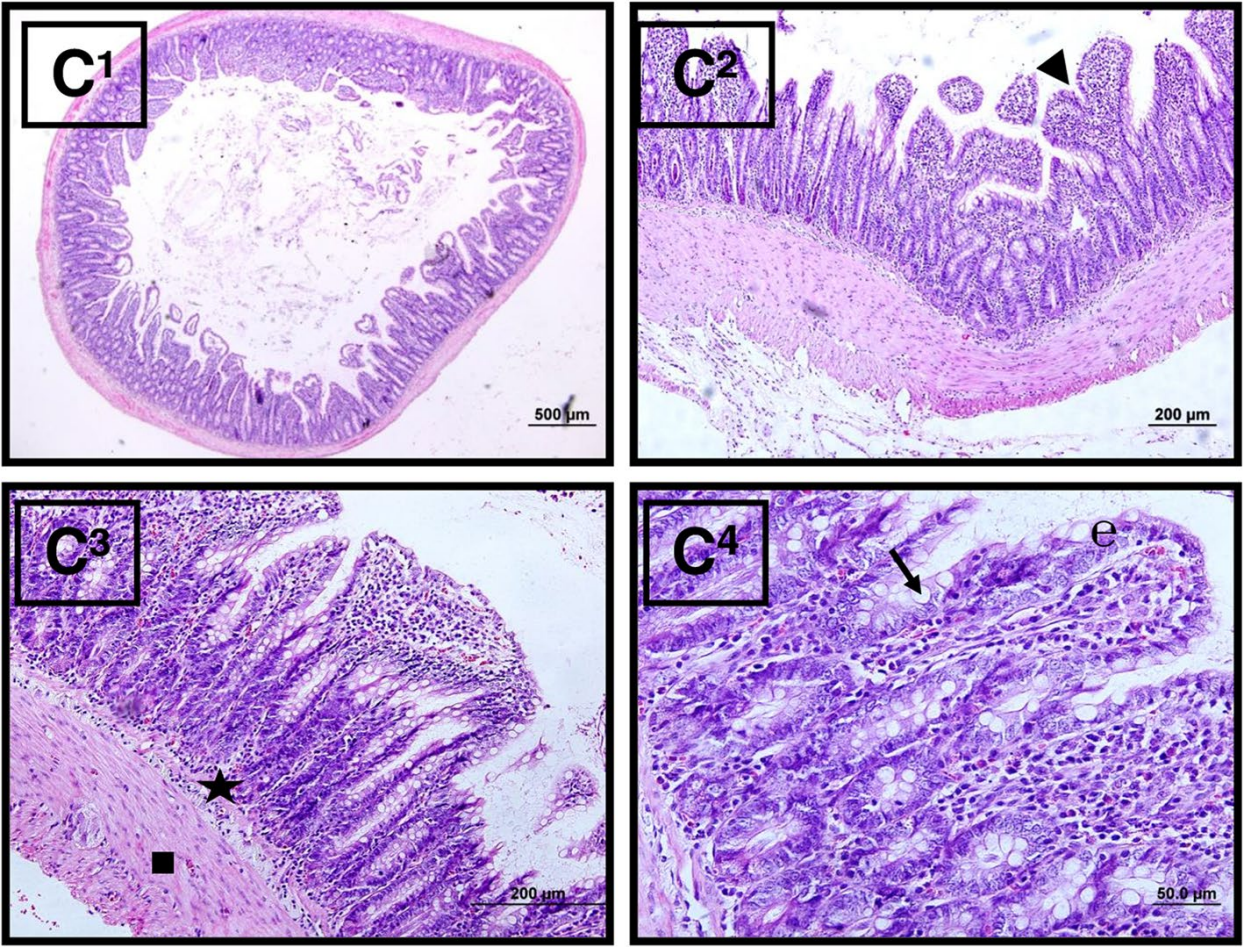
Microscopic view of the IIR + L group tissue samples. Epithelial layer (**e**), submucosal layer (black star), tunica muscularis (square), and goblet cells (right arrow) are shown. Hematoxylin–eosin (H&E) stain with amplifications of C1: × 4, C2: × 10, C3: × 20, C4: × 40)

The villi structures were better preserved and there was less mononuclear cell infiltration, and vascular dilatation and hemorrhage were less common in the lamina propria when compared to the IIR group (Fig. 8).

## Discussion

The study was designed to show the effects of early administration of levosimendan on intestinal damage after ischemia–reperfusion in an experimental intestinal injury-reperfusion model. Our study revealed that levosimendan reduces small intestine damage in terms of Chiu score.

Intestinal ischemia and reperfusion lead to the formation of inflammatory cytokines and reactive oxygen species [12]. Cytotoxic events disrupt the barrier function of the intestine and cause toxic products to pass into the systemic circulation, affecting the organs such as the kidneys, liver, and heart leading to multiple organ failure (MOF) [13]. There are basic treatment methods that aim at reducing ischemia–reperfusion damage and preventing the development of MOF [14]. For this purpose, ischemic preconditioning, treatment with antioxidant agents, nitric oxide (NO) applications, anti-complement therapy, and pharmacological preconditioning caused by various agents have been used [15].

Mallick et al. created their experimental models by performing 30 min ischemia and 120 min reperfusion [16]. In our study, longer ischemia time was aimed and 60-min ischemia and 120-min reperfusion model used by Özkaya [9] and Zhang [17] were applied.

Özkaya et al. [9] investigated the effects of pre-treatment levosimendan on small bowel damage in the rat IIR model. The investigators administered a loading dose of 12 µg.kg^−1^ levosimendan before ischemia followed by a maintenance infusion of 0.2 µg.kg^−1^.min^−1^ during 60 min of ischemia. The recommended clinical dose of the agent was reported as 0.05–0.2 µg.kg^−1^.min^−1^ infusion after 6–24 µg.kg^−1^ loading dose [18]. In our study, we administered a maintenance infusion dose of 0.2 µg.kg^−1^.min^−1^ following a 12 µg.kg^−1^ during a 10-min loading dose, a dosage whose efficacy has been shown in two separate experimental studies [9, 10].

Ahmetova et al. [19] investigated the effect of levosimendan in the hepatic IR model and reported a decrease in MAP measurements at the 15th and 45th minute of reperfusion in the IR group. It has been reported that the effect of levosimendan peaks within 10–30 min following the loading dose, and in addition to its positive inotropic effects, levosimendan opens the ATP-dependent K + channels in myocytes and vascular smooth muscle cells, causing vasodilation in the systemic vascular bed leading to hypotension [20]. Oldner et al. suggested that levosimendan infusion initiated before endotoxin led to a 37% decrease in systemic vascular resistance index and approximately 22% decrease in the mean arterial pressure [7]. In our study, there was no decrease in MAP-8 and MAP-9 after 10 min of 12 µg.kg^−1^ loadings and 50 min of 0.2 µg.kg^−1^.min^−1^ levosimendan infusion, respectively. During these time periods, the expected difference between all 3 groups did not occur, and we observed that the blood pressure values of the IIR + L group were similar to the sham group. This result suggested that levosimendan enhances cardiac performance in IIR [21], preserving systemic blood pressure despite reperfusion.

It has been reported that free oxygen radicals and hydrogen peroxide, which increase as a result of intestinal IR damage, initiate lipid peroxidation and protein damage, leading to cell apoptosis and tissue necrosis. MDA and thiobarbituric acid reactive substances (TBARS) are released as a result of lipid peroxidation [22]. In our study, the MDA level, which is the end product of lipid peroxidation, was measured as an indicator of free oxygen radical formation in tissues. Although there was no difference between the groups when compared statistically, we observed that MDA levels were the highest in the IIR group and lowest in the *sham* group, and MDA levels in the IR + L group were less than in the IR group. In an experimental study by Özkaya et al. [9], levosimendan was applied before the IR period, and tissue TBARS levels were found to be significantly higher in the IR group when compared to the sham and IR + L groups. This result shows that levosimendan administered as a pretreatment reduces the lipid peroxidation that occurs after intestinal ischemia–reperfusion. Investigating the effect of levosimendan on oxidative stress in myositis culture, Maytin et al. [23] found that the drug opened K-ATP channels in the clinical therapeutic dose range and protected the cell from apoptosis caused by hydrogen peroxide. Apoptosis has an important role in ischemia–reperfusion injury [24]. In our study, although the administration of levosimendan after reperfusion was shown to contribute to a decrease in lipid peroxidation, this could not be shown statistically.

Various scores are used in the histopathological classification of intestinal IR injury. Chiu et al. [11], Park et al. [25], and Hierholzer et al. [26] have all reported different scoring systems.

In our study, we found that the villi structure in the small intestines was severely damaged in the IIR group, and ulceration and mononuclear cell infiltration in the lamina propria were significantly increased, and therefore, the Chiu score was significantly higher when compared to the scores of the sham and IIR + L groups. Although there was a difference in the Chiu score of the IIR + L group when compared to the sham group, the presence of hemorrhage, ulceration, and mononuclear cell number were very similar to the sham group in light microscopic imaging. Similar histopathological findings were found by Özkaya et al. [9], Mallick et al. [16], Topaloğlu et al. [12], and Zhang et al. [17] who used levosimendan, pyrrolidine dithiocarbamate, prostaglandin E_2_ (PGE2), and glucagon-like peptide 2 (GLP-2) as pre-medication before IR damage, respectively.

Levosimendan increases intestinal perfusion and oxygenation by positively affecting splanchnic and mesenteric blood flow [27, 28]. Garcia-Septiem et al. [27] demonstrated that levosimendan pretreatment improved portal blood flow, intestinal mucosa oxygenation, and pulmonary functions in pigs in a septic shock model created with intravenous *Escherichia coli* (*E. coli*). In our study, the Chiu score was found to be statistically significantly lower in the levosimendan group when compared to the IIR group. We believe this was caused by the decrease in splanchnic resistance, the increase in regional blood flow, and mucosal oxygenation. Emphasizing that levosimendan is superior to milrinone and dobutamine in increasing gastric mucosal oxygenation, it has been recommended that levosimendan be used as an alternative in patients with splanchnic ischemia risk [8, 29]. Özkaya et al. [9] reported that levosimendan infusion initiated before ischemia decreased neutrophil accumulation, lipid peroxidation, and the Chiu score in the intestines. However, clinically, it can be difficult to know the exact time of onset of ischemia. The authors concluded that further experimental studies investigating the initiation time and duration of the application are required for the results of the research to gain relevant clinical significance.

### Limitations

Although levosimendan infusion administered for 1 h from the 2nd hour of intestinal reperfusion decreased the histological damage score without causing adverse hemodynamic effects, we were unable to demonstrate its effect on lipid peroxidation. Also, this study did not evaluate myeloperoxidase, inflammatory cytokines, and adhesion molecules showing neutrophil adhesion. It would be appropriate to support the results of this experimental study with comprehensive biochemical parameters and drug administration starting from the reperfusion process.

## Conclusion

Levosimendan leads to a decrease in intestinal damage although it did not affect lipid peroxidation and MAP when administered after reperfusion in an experimental intestinal IR model. Administration of levosimendan after intestinal reperfusion may provide clinical benefit.

## Data Availability

Not applicable.
